# Treatment landscape from first‐ to third‐line therapy and quality of life data of patients with pancreatic cancer from the prospective German PARAGON (Platform for Outcome, Quality of Life, and Translational Research on Pancreatic Cancer) registry (IKF‐PARAGON study)

**DOI:** 10.1002/ijc.70280

**Published:** 2025-12-13

**Authors:** Thorsten O. Goetze, Salah‐Eddin Al‐Batran, Ruediger Liersch, Lars Scheuer, Thomas Goehler, Ulrich Kaiser, Claudio Denzlinger, Stephan Brandl, Daniel Pink, Jens Uhlig, Michael Maasberg, Maria Loose, Marina Schaaf, Disorn Sookthai, Bianca Zäpf, Claudia Pauligk, Timursah Habibzade, Ralf‐Dieter Hofheinz, Christine Koch, for the AIO Study Group

**Affiliations:** ^1^ University Cancer Center (UCT) Krankenhaus Nordwest Frankfurt am Main Germany; ^2^ University Cancer Center (UCT) Goethe University Frankfurt Frankfurt am Main Germany; ^3^ The Frankfurt Institute of Clinical Cancer Research, IKF Frankfurt am Main Germany; ^4^ GEHO, Practice for Hematology and Medical Oncology Münster Germany; ^5^ Onkologische Schwerpunktpraxis Speyer Speyer Germany; ^6^ Onkozentrum Dresden/Freiberg/Meißen Dresden Germany; ^7^ Department for Hematology, Oncology and Immunology St. Bernward Krankenhaus Hildesheim Germany; ^8^ Department for Hematology, Oncology, Cell Therapy and Palliative Care Marienhospital Stuttgart Stuttgart Germany; ^9^ Überörtliche Gemeinschaftspraxis, Schwerpunkt Hämatologie, Onkologie und Palliativmedizin Hamburg Germany; ^10^ Klinik für Hämatologie Onkologie und Palliativmedizin, Sarkomzentrum Berlin‐Brandenburg, HELIOS Klinikum Bad‐Saarow Bad Saarow Germany; ^11^ Praxis für Hämatologie und Onkologie Naunhof Germany; ^12^ Internistisches Haus‐ und Facharztzentrum Mayen Germany; ^13^ II. Medizinische Klinik Universitätsmedizin Mannheim Mannheim Germany; ^14^ Department of Gastroenterology Frankfurt University Clinic, Goethe University Frankfurt Frankfurt Germany

**Keywords:** pancreatic cancer, quality of life, treatment sequence

## Abstract

Pancreatic cancer (PCA) is the third leading cause of cancer‐related death in Europe. Despite recent therapeutic advances, patients experience rapid health deterioration. Based on previous results, the Platform for Outcome, Quality of Life, and Translational Research on Pancreatic Cancer—PARAGON (NCT04119362) was initiated to investigate the whole life cycle of PCA patients. Between November 2019 and October 2021, 469/479 screened patients were enrolled in 46 sites. Demographic, clinical, and quality of life (QoL) data were collected. The treatment landscape was depicted using Sankey diagrams. Median overall survival (mOS) for all patients in first line was 10.6 months (95% confidence interval [CI], 9.2–11.7 months). With mFOLFIRINOX as first‐line treatment, mOS was 11.3 months (95% CI, 8.6–13.5 months), with gemcitabine/nab‐paclitaxel 10.5 months (95% CI, 8.3–12.9 months). The mean Global Health Status for patients that proceeded from first to second line did not substantially deteriorate during first line. Predictive variables for proceeding from first to second‐line therapy were reasons for ending first‐line treatment (patient's wish, toxicity, and progressive disease) and age. In summary, we were able to show in detail patient flows and QoL data throughout all therapy lines, which will help to further understand the major clinical checkpoints of the disease.

## What's New?

Pancreatic cancer often is diagnosed at advanced stages. As a result, treatment regimens frequently are aggressive, with substantial side effects that impact quality of life (QoL). This study examined the treatment landscape, from neoadjuvant therapy to palliative care, in the context of QoL among pancreatic cancer patients in Germany. Most patients initiated therapy with palliative intent. The predominant first‐line regimens were gemcitabine/nab‐paclitaxel, modified FOLFIRINOX, and gemcitabine monotherapy, with notable differences in treatment efficacy. QoL, although lower than the general population, was maintained during first‐line treatment. Treatment termination was influenced by factors such as age, toxicity, disease progression, and patient wish.

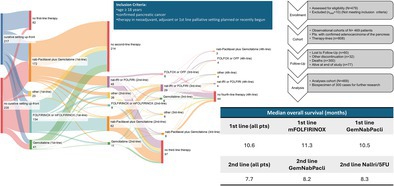

**Abbreviations**
AGEO: Association des gastro‐entérologues oncologues
CI: confidence interval
ECOG: Eastern Cooperative Oncology Group
eCRF: electronical case report form
EORTC QLQ‐C30 questionnaire: European Organization for Research and Treatment of Cancer (EORTC) Quality of Life Questionnaire‐Core 30 Items Scale (QLQ‐C30)
FOLFIRI: fluorouracil leucovorine, irinotecan
FOLFOX: fluorouracil leucovorine, oxaliplatin
FOLFIRINOX: fluorouracil, leucovorin, irinotecan and oxaliplatin
GHS/QoL: Global Health Status/Quality of life
HR: hazard ratio
mFOLFIRINOX: modified fluorouracil leucovorine, irinotecan, oxaliplatin
mPFS: (median) progression free survival
NalIri: nanoliposomal‐irinotecan
NALIRIFOX: nanoliposomal‐irinotecan, fluorouracil leucovorine, oxaliplatin
OFF: fluorouracil leucovorine, oxaliplatin
ORR: overall response rate
OS: overall survival
PARAGON: Platform for Outcome, Quality of Life, and Translational Research on Pancreatic Cancer
PCA: pancreatic cancer
PDAC: pancreatic ductal adenocarcinoma
PFS: progression‐free survival
QoL: quality of life
TTD: time to deterioration

## 1 INTRODUCTION

Pancreatic cancer (PCA) is the third leading cause of cancer‐related death in the United States and European countries.^1^ It is one of the most lethal cancers with a 5‐year survival rate of 10%. Surgical resection is currently the only potentially curative treatment option. However, due to the lack of effective screening possibilities, most patients present with locally advanced or metastatic, thus unresectable disease at diagnosis.

Within the past decades, two combination treatment regimens emerged as first‐line therapy for patients with advanced PCA, for whom before mainly gemcitabine was available. The global MPACT phase 3 trial demonstrated an improved overall survival (OS) for patients with metastatic PCA treated with gemcitabine plus nab‐paclitaxel compared to gemcitabine monotherapy (8.5 vs. 6.7 months, hazard ratio [HR] 0.72, *p* < .001).^2^ In addition, treatment with the triplet chemotherapy regimen FOLFIRINOX (5‐fluorouracil [5‐FU], leucovorin, irinotecan, and oxaliplatin) was shown to further prolong the OS compared to gemcitabine monotherapy (11.4 vs. 6.8 months, HR 0.57, *p* < .001)^3^ in a pivotal French Phase 3 trial. Due to the higher toxicity profile associated with the FOLFIRINOX treatment and the original inclusion criteria from the clinical trial that excluded patients older than 75 years, this regimen is usually applied in fit patients younger than 75 years, whereas gemcitabine plus nab‐paclitaxel is used for patients with mediocre performance, comorbidities, or older age. Besides, a recent meta‐analysis that included data on 2581 patients, including 383 patients treated with anoliposomal‐irinotecan, fluorouracil leucovorine, oxaliplatin (NALIRIFOX), 433 patients treated with FOLFIRINOX, and 1756 patients treated with gemcitabine plus nab‐paclitaxel, underscored that the toxicity profile among the different regimens needs to be considered rather than the sheer incidence, which was comparable.^4^ After progression under first‐line gemcitabine‐based treatment, second‐line therapy with nanoliposomal irinotecan combined with 5‐FU is recommended based on the results of the NAPOLI‐1 trial.^5^ Some prospective trials that directly compared first‐line treatment with the doublet versus the triplet regimen (e.g., GENERATE,^6^ PASS‐01,^7^ NAPOLI‐3^8^) have so far shown conflicting data on quality of life (QoL) as well as efficacy. Therefore, real‐world evidence from prospective registries is needed to guide treatment decisions, which must take all aspects of a patient's life into account. Also, most clinical trials focus on one specific line of treatment only and do not depict the complete landscape including prior and subsequent therapies as well as adjustments and changes made during the treatment period.

Based on our previous works investigating QoL in cancer patients,^8^, ^9^ in 2014, we initiated the QOLIXANE study (NCT02691052) that evaluated the health‐related QoL of patients with metastatic PCA receiving first‐line gemcitabine plus nab‐paclitaxel. Overall, this study showed that QoL rapidly declined for most patients, reflecting the aggressive nature of PCA. However, QoL stabilization appeared to be possible for a subset of patients receiving gemcitabine plus nab‐paclitaxel in first line.^9^ Based on these results, this non‐interventional, prospective, multicenter study evolved into the Platform for Outcome, Quality of Life, and Translational Research on Pancreatic Cancer—PARAGON (NCT04119362). This prospective registry for pancreatic ductal adenocarcinoma in Germany investigated the whole life cycle of PCA patients from neoadjuvant to last line situations, depicting the different outcomes of the patients in their different stages of disease. The aim of PARAGON was to generate further information on the QoL and care of these patients and to map the complete life cycle of a large cohort of real‐life PCA patients. In this paper, we present the first results from those patients receiving neoadjuvant, adjuvant, and palliative treatment, illustrating patient flows from diagnosis across the lines, as well as data on QoL.

## 2 PATIENTS AND METHODS

### 2.1 Study design and participants

The PARAGON study (IKF‐642) is a multicenter, prospective, non‐interventional, German registry with biobanking. Main eligibility criteria comprised age 18 years and older, histologically, or cytologically confirmed PCA and for whom therapy in the neoadjuvant, adjuvant, or first‐line palliative setting was planned or had recently begun (within 14 days of enrollment).

### 2.2 Observations

At the time of study entry, relevant clinical data, including demographic information, Eastern Cooperative Oncology Group (ECOG) performance status, age, comorbidities, tumor anamnesis, and previous treatment were captured in an electronic case report form (eCRF). After the start of therapy, available treatment‐related parameters, objective response (if available), and dates of progression and death were documented. QoL data were assessed using the validated European Organization for Research and Treatment of Cancer (EORTC) Quality of Life Questionnaire‐Core 30 Items Scale (QLQ‐C30) (EORTC QLQ‐C30 questionnaire) prior to the beginning of every therapy line, every 8 weeks during and at the end of treatment. In addition, the patients were asked to complete a short questionnaire (four items) on their self‐perceived performance status. Owing to the observational character of the study, no instructions were made to the study participants concerning the type and time of measurements or treatment during the study.

### 2.3 Study objectives

The primary objective of PARAGON was to determine the treatment landscape in Germany and the course of QoL throughout therapy for patients with PCA. The secondary objectives included assessment of progression‐free survival (PFS) and OS for all treatment lines as well as OS rates for neoadjuvant/adjuvant treatment. Prospects of PCA research, for example, for the identification of clinical and biological prognostic factors, are supported by PARAGON's biobank accessibility.

### 2.4 Statistical analysis

Since this was a descriptive study, there was no formal calculation of sample size. All parameters were evaluated in an explorative or descriptive manner, providing means, medians, interquartile and total ranges, standard deviations and/or confidence intervals (CI), counts and proportions, or Kaplan–Meier curves, as appropriate for the respective data types. In general, all analyses presented, and calculations performed are based on the data available for each item (observed case analyses). Missing data were not extrapolated. The number of missing values was computed. For the time‐to‐event variable OS, the Kaplan–Meier method was used. OS was defined as time from initiation of therapy to the date of death of any cause. If no event was observed (e.g., lost to follow‐up), OS was censored at the day of last subject contact. The time to deterioration (TTD) of QoL is defined as the period between baseline assessment and the first observation of a decreased score by more than 10 points. If no such deterioration is observed, the date of the final questionnaire is considered a censored observation. EORTC QLQ‐C30 questionnaires were analyzed according to the EORTC QLQ‐C30 scoring manual. Supplemental questions, which were not validated, were evaluated individually. Data analysis was generated using SAS software, Version 9.4 of the SAS‐System for Windows. Sankey diagrams were built using specifically extracted and formatted data with the free software sankeyMATIC (https://sankeymatic.com/build/).

## 3 RESULTS

### 3.1 Demographics

**FIGURE 1:**
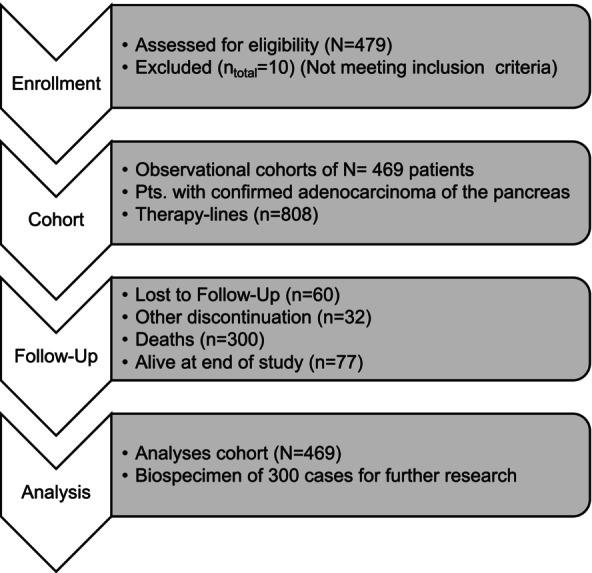
CONSORT diagram.

Between November 2019 and October 2021, 479 patients were screened for eligibility and 469 patients were enrolled (Figure 1) in 46 of the 53 participating sites (seven sites were activated but did not enroll patients). The study population comprised 53% male patients, a median age of 69 years (range, 31–90 years). Of the 46 sites, *n* = 3 (6.5%) were university hospitals, *n* = 20 (43.5%) community hospitals and *n* = 23 (50%) private practices (smaller offices and ambulatory health centers). Of the enrolled patients, nine patients died within 17–86 days prior to any cancer treatment. Localization of the primary tumor as well as metastases reflects the known incidence of anatomical locations with most cancers occurring in the pancreatic head.^10^ Concomitant diseases were reported for 380/469 patients (81.0%) and reflect the spectrum of diseases in an elderly real‐world population.

### 3.2 Treatment landscape

**FIGURE 2:**
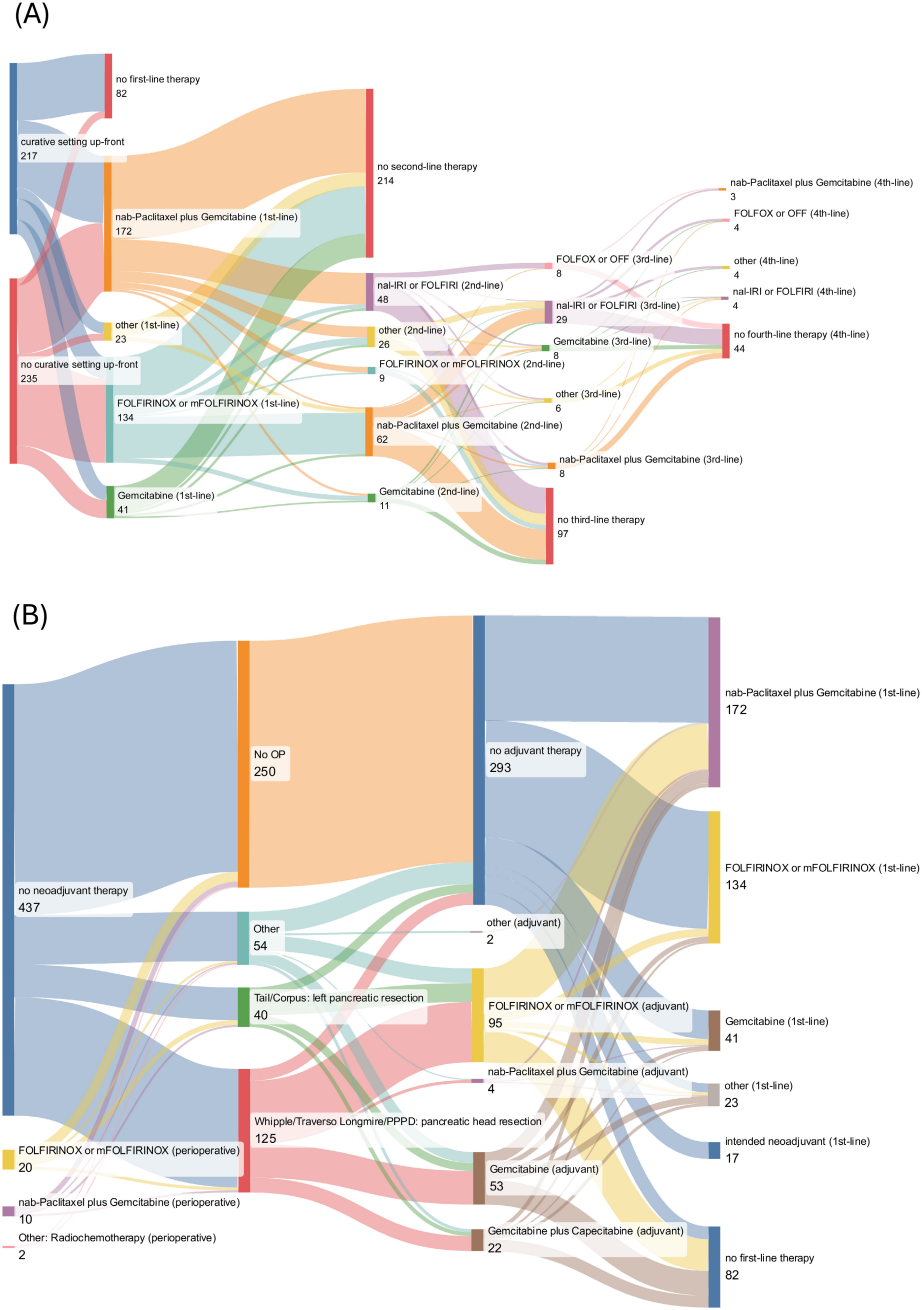
(A) Sankey diagram, all patients; other: Total pancreatectomy or other surgeries (explorative laparotomy, *n* = 1; total pancreatectomy, *n* = 26; other, *n* = 27). (B) Sankey diagram, patients with primarily curative versus palliative treatment intention at the time of treatment start. Patients with intended neoadjuvant treatment not included. “Other” first line: nanoliposomal‐irinotecan (NalIri) or fluorouracil leucovorine, irinotecan (FOLFIRI), *n* = 10; gemcitabine and capecitabine, *n* = 5; fluorouracil leucovorine, oxaliplatin (FOLFOX) or fluorouracil leucovorine, oxaliplatin (OFF), *n* = 3; paclitaxel and gemcitabine, *n* = 2; capecitabine, *n* = 1; gemcitabine and erlotinib, *n* = 1; chemoradiotherapy, *n* = 1; “other” second line: chemoradiotherapy, *n* = 6; FOLFOX or OFF, *n* = 6; capecitabine, *n* = 4; gemcitabine and erlotinib, *n* = 3; radiotherapy, *n* = 1; 5‐fluorouracil and folinic acid, *n* = 1; gemcitabine and capecitabine, *n* = 1; oxaliplatin and capecitabine, *n* = 1; gemcitabine and cisplatin, *n* = 1; stereotactic ablative radiotherapy, *n* = 1; carboplatin, *n* = 1; methotrexate, *n* = 1; “other” third line: cisplatin, *n* = 1; (m)FOLFIRINOX, *n* = 1; capecitabine and oxaliplatin, *n* = 2; irinotecan mono, *n* = 1; gemcitabine and cisplatin, *n* = 1; gemcitabine and erlotinib, *n* = 1; “other” fourth line: gemcitabine, *n* = 1; gemcitabine and cisplatin, *n* = 1; 5‐fluorouracil, *n* = 1. FOLFIRINOX, fluorouracil, leucovorin, irinotecan, and oxaliplatin.

Patients were divided into two groups based on the primary intention of their therapy, that is, curative or palliative intent. Due to the nature of the disease, the primary intention of treatment had to be corrected in several patients during their disease (e.g., from neoadjuvant to palliative). Therefore, we decided to depict the treatment landscape as Sankey diagrams (Figure 2A,B) with the primary therapeutic intention as a starting point. One hundred and seventy‐six out of 469 patients (37.5%) received adjuvant therapy (FOLFIRINOX, *n* = 95; gemcitabine, *n* = 53; gemcitabine/capecitabine *n* = 22; nab‐paclitaxel/gemcitabine, *n* = 4; other therapies, *n* = 2), whereas 34 (7%) received surgery in a curative setting without systemic chemotherapy administered perioperatively. Thirty‐two out of 469 patients (7%) received perioperative therapy (FOLFIRINOX, *n* = 20; nab‐paclitaxel/gemcitabine, *n* = 10; radiochemotherapy, *n* = 2).

Three hundred and eighty‐seven out of 469 patients (83%) received systemic first‐line therapy at any point in time (FOLFIRINOX, *n* = 134; nab‐paclitaxel/gemcitabine, *n* = 172; gemcitabine, *n* = 41; other, *n* = 23). This includes patients with upfront systemic treatment (e.g., with locally advanced, unresectable tumor or synchronous metastases) as well as 17 patients with intended neoadjuvant treatment who needed to switch to a palliative setting, for example, due to progression. Of the 387 patients who received first‐line treatment, 214 patients (55%) did not proceed to second‐line treatment, and of the 166 patients in second line, 95 (57%) did not proceed to further lines. In second line, patients received mainly nab‐paclitaxel/gemcitabine (*n* = 69), (liposomal) irinotecan‐based treatment (*n* = 49) or gemcitabine (*n* = 13) as well as other, individual therapies (*n* = 35). In third line, patients were treated with (liposomal) irinotecan‐ or oxaliplatin‐based treatments (*n* = 28) or gemcitabine‐based treatments (*n* = 16). Taken together, only a minority of patients in this real‐world setting was able to receive more than one line of treatment, highlighting the need for QoL assessments and treatment adjustments.

To understand the relationship between various patient characteristics and the likelihood of proceeding to second‐line treatment after the end of therapy in the first line, we used logistic regression. The outcome variable in this analysis is binary, indicating whether a patient proceeded to second line after ending first‐line therapy. The predictor variables we considered include age, sex, ECOG at baseline, and reason for the end of first‐line therapy. We included only patients that stopped first‐line therapy for reasons other than death in this analysis (*n* = 314, one patient excluded due to missing data).

The reason for the end of first‐line therapy was identified as a significant predictor variable for proceeding to second line. Patients who end first‐line therapy at their own wish have a smaller likelihood to proceed to second‐line therapy than patients who have to end first‐line therapy due to progression (overall response rate (ORR) 15.16; 95% CI, 6.16, 37.32; *p* < .0001), toxicity (ORR 6.48; 95% CI, 2.39, 17.6; *p* = .1897) or other reasons (ORR 4.66; 95% CI 1.86, 11.7; *p* = .9705). Additionally, a trend for age as a predictor variable was observed. Patients younger than 70 years have a greater likelihood to proceed to second line than patients aged 70 years or older (ORR 1.57; 95% CI, 0.95, 2.61; *p* = .0815).

### 3.3 Survival

**FIGURE 3:**
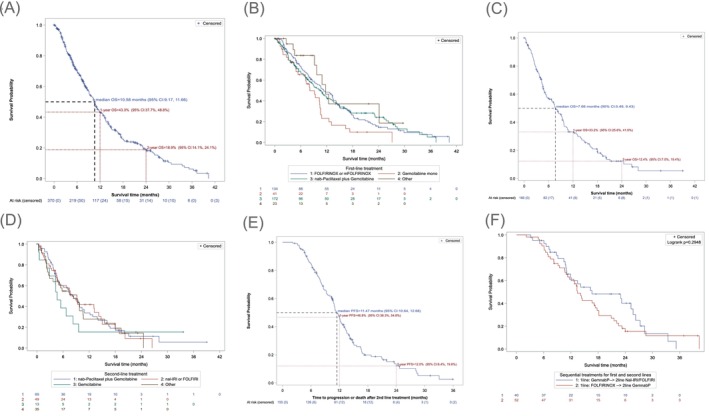
(A) Overall survival (OS), all patients receiving any systemic first‐line treatment excluding patients with intended neoadjuvant treatment. Kaplan–Meier analysis. (B) Overall survival, all patients receiving any systemic first‐line treatment excluding patients with intended neoadjuvant treatment (other: NalIri or FOLFIRI, *n* = 10; gemcitabine and capecitabine, *n* = 5; FOLFOX or OFF, *n* = 3; paclitaxel and gemcitabine, *n* = 2; capecitabine, *n* = 1; gemcitabine and erlotinib, *n* = 1; chemoradiotherapy, *n* = 1); Kaplan–Meier analysis. (C) OS, all patients receiving any systemic second‐line treatment excluding patients with intended neoadjuvant treatment. Kaplan–Meier analysis. (D) OS after second‐line treatment, all patients receiving any systemic second‐line treatment excluding patients with intended neoadjuvant treatment (other: capecitabine, *n* = 3; FOLFIRINOX (fluorouracil, leucovorin, irinotecan, and oxaliplatin) or mFOLFIRINOX, *n* = 9; FOLFOX or OFF, *n* = 6; gemcitabine and capecitabine, *n* = 1; gemcitabine and erlotinib, *n* = 3; chemoradiotherapy, *n* = 6; 5‐fluorouracil, *n* = 1; capecitabine and oxaliplatin, *n* = 1; carboplatin, *n* = 1; gemcitabine and cisplatin, *n* = 1; methotrexate, *n* = 1; radiotherapy, *n* = 1; stereotactic ablative radiotherapy, *n* = 1). Kaplan–Meier analysis. (E) Median progression‐free survival (PFS) 1 + 2 (i.e., combined PFS after progression on second‐line treatment) for all patients (*n* = 155) that received any second‐line treatment. Kaplan–Meier analysis. (F) OS, all patients receiving sequential treatment with gemcitabine and nab‐paclitaxel in first line and NalIri/FOLFIRI in the second line versus patients receiving FOLFIRINOX in the first line and gemcitabine and nab‐paclitaxel in the second line. Kaplan–Meier analysis. CI, confidence interval.

The median follow‐up time of surviving patients was 7.6 months (range, 0–40.0). In first line, median overall survival (mOS) was 10.6 months (95% CI; 9.2, 11.7) for all patients. Patients receiving mFOLFIRINOX as first‐line treatment had an mOS of 11.3 months (95% CI; 8.6, 13.5), whereas patients being treated with gemcitabine/nab‐paclitaxel in the first line showed an mOS of 10.5 months (95% CI; 8.3, 12.9; Figure 3A,B). In second line, mOS was 7.7 months (95% CI; 5.5, 9.4) for all patients. Patients receiving gemcitabine/nab‐paclitaxel as second‐line treatment had an mOS of 8.2 months (95% CI; 5.2, 10.4), whereas patients being treated with gemcitabine mono showed an mOS of 4.5 months (95% CI; 1.2, 8.3) and (nanoliposomal) irinotecan/5‐FU of 8.3 months (95% CI; 4.6, 13.2; Figure 3C,D). We specifically investigated patients that received non‐canonical treatment sequences: median PFS 1 + 2 (i.e., combined PFS after progression on second‐line treatment) for all patients (*n* = 155) that received any second‐line treatment was 11.47 months, the PFS rate at 12 months was 47%. In patients that received gemcitabine/nab‐paclitaxel as first line and NalIri/FOLFIRI as second‐line treatment (*n* = 40), (median) progression free survival (mPFS) (again PFS 1 + PFS 2) was 13.47 months with a PFS rate at 12 months of 53%; mOS in this group was 16.7 months. Treatment with mFOLFIRINOX in the first line and gemcitabine/nab‐paclitaxel in the second line (*n* = 52) led to an mPFS (PFS 1 + PFS 2) of 11.27 months and a 12‐month PFS rate of 47% (Figure 3E,F), while the mOS in this group was 13.4 months. OS in third line was 4.3 months (95% CI; 2.7, 8.1).

### 3.4 Quality of Life

The ECOG performance status was self‐reported by the patients and rated by the treating physicians. Physicians tended to rate the patient's ECOG performance score better than the patients themselves with more patients rated as 0/1 from the physicians' viewpoints (physician‐based ECOG 0/1: *n* = 358; patient‐based ECOG 0/1: *n* = 281; identical assessment in 38% of cases).

Baseline EORTC QLQ‐C30 questionnaires were available from 335/370 (90.5%) patients in a first line and 119 out of 167 (71.3%) patients in a second‐line setting. The rate of first line patients with baseline plus at least one on‐study questionnaire was 46.3% for patients receiving gemcitabine, 53.5% for gemcitabine/nab‐paclitaxel and 63.4% for (m)FOLFIRINOX, respectively.

**FIGURE 4:**
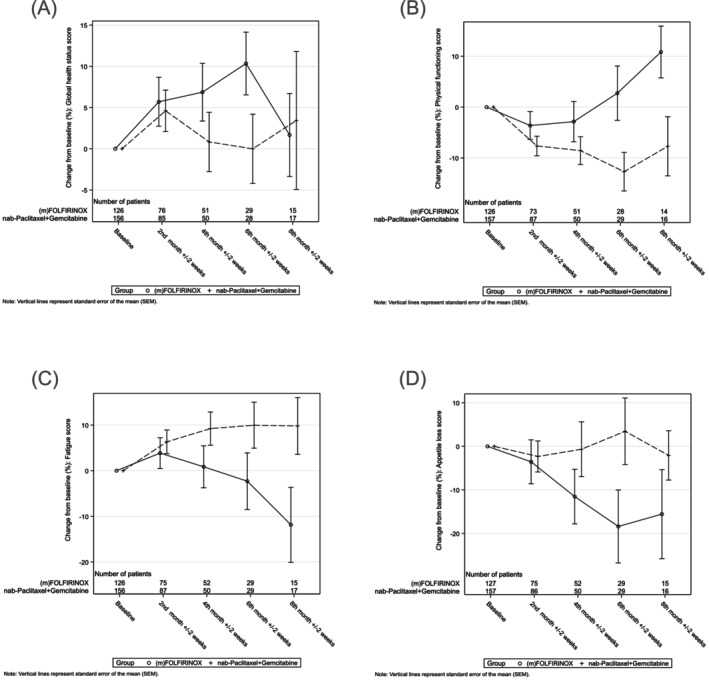
(A) Global Health Status, mean change from baseline, according to treatment group (first line). (B) Physical functioning score, mean change from baseline, according to treatment group (first line). (C) Fatigue score, mean change from baseline, according to treatment group (first line). (D) Appetite loss score, mean change from baseline, according to treatment group (first line). FOLFIRINOX, fluorouracil, leucovorin, irinotecan, and oxaliplatin.

The mean baseline Global Health Status was 47.7 ± 22.03 in patients treated with (m)FOLFIRINOX (*n* = 126) and 45.8 ± 22.96 in patients treated with gemcitabine/nab‐paclitaxel (*n* = 156; Figure 4A). For patients with baseline plus at least one on‐study questionnaire (*n* = 204), the difference from baseline after 2, 4, 6, and 8 months (each ±2 weeks) for the EORTC QLQ‐C30 subscales was calculated. The Global Health Status remained almost unchanged in the doublet and the triplet regimens; however, patient numbers declined over time, which limits the interpretation. Keeping this in mind and focusing on single items from the questionnaire, several items stand out: the physical functioning score drifts apart with a numerical advantage over time for patients in the triplet regimen, while the fatigue and appetite loss scores decrease (meaning less symptom burden), also in favor of the triplet group (Figure 4B–D). At the start of second‐line treatment, the mean Global Health Status Score for all evaluable patients from first line that progressed to second line (*n* = 112) was 48.2 ± 20.9, thus not significantly different from the start of first‐line treatment.

Other items, such as role functioning, emotional functioning, financial problems, cognitive and social scores, nausea/vomiting, pain, dyspnea, insomnia, constipation, and diarrhea did not substantially change over time or differ between the groups (data not shown).

**FIGURE 5:**
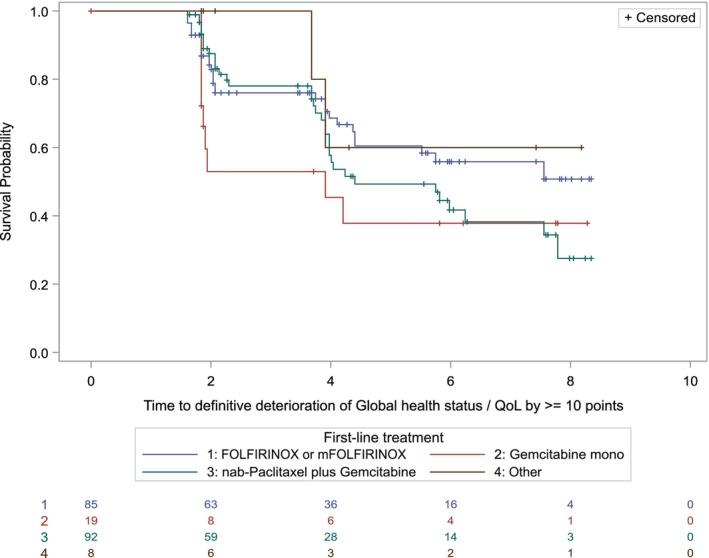
Time to definitive deterioration of Global Health Status by ≥10 points according to treatment group. FOLFIRINOX, fluorouracil, leucovorin, irinotecan, and oxaliplatin; QoL, quality of life.

The median TTD of Global Health Status/Quality of life (GHS/QoL) score by ≥10 points was 5.8 months in the first line as well as 6.2 months in the second‐line situation (Figure 5). In the first line situation, the median TTD was about as long as the mPFS of 6.0 months for all patients (95% CI; 5.3, 7.0). In contrast, in second line patients the median TTD was with 6.2 months (95% CI; 5.5, ne) roughly double as long as the 3.75 months mPFS (95% CI; 2.96, 4.76). Of note, from initially *n* = 166 patients with baseline questionnaires at the start of second‐line treatment, a baseline and a post‐baseline assessment were available from *n* = 71 patients for analysis.

## 4 DISCUSSION

The multicenter, prospective PARAGON register enrolled 469 PCA patients who were scheduled for or had recently started neoadjuvant, adjuvant, or first‐line therapy, and followed the course of disease of these patients for up to almost 4 years. The study population comprised 53% male, a median age of 69 years, with ECOG 0 to 1 in 76% at baseline. Compared to demographic data from the German Cancer Registry for the years 2019/2020, the median age in our group was slightly younger than in the registry, where the median age at first diagnosis was 76 years for female and 72 years for male patients, while the distribution between the sexes was comparable.^11^

Of the 469 enrolled patients, the majority started treatment with palliative intent. The three most common first‐line therapies were gemcitabine/nab‐paclitaxel, (modified) FOLFIRINOX and gemcitabine monotherapy, with differences in efficacy between regimens.

Compared to results from the German TPK clinical cohort study, in which treatment algorithms and survival of 1174 patients with locally advanced, inoperable, or metastatic PCA were analyzed,^12^ we saw some differences in our smaller cohort: Of all patients receiving first‐line chemotherapy in the TPK trial, only 40% and 13% received second‐ or third‐line treatment, respectively. In our trial, slightly more participants advanced to further lines, although it was still less than half of the patients. One possible explanation could be the enrollment period, which was earlier in the TPK trial (2014–2017) than in PARAGON (November 2019–October 2021), reflecting changing treatment options.

The results of the OS subgroup analysis for the first‐line treatment reflect well the data of the phase 3 trials for FOLFIRINOX (PRODIGE)^3^ and nab‐paclitaxel/gemcitabine (MPACT),^2^ with the results for gemcitabine/nab‐paclitaxel being slightly advantageous in our registry: FOLFIRINOX, 11.33 months (PRODIGE, 11.1 months) and nab‐paclitaxel/gemcitabine, 10.45 months (MPACT, 8.5 months). However, the different nature of data generation does not allow a formal comparison here.

A recent meta‐analysis by Nichetti et al.^4^ that included 383 patients treated with NALIRIFOX, 433 patients treated with FOLFIRINOX, and 1756 patients treated with nab‐paclitaxel/gemcitabine showed a mOS of 10.4 months for patients treated with nab‐paclitaxel/gemcitabine, compared to 11.7 months for FOLFIRINOX and 11.1 months for NALIRIFOX; taken together, the results from the registrational trials were confirmed, although differences in the safety profile were evident with more patients suffering from diarrhea in the NALIRIFOX group.

In second line, data is available from the registrational NAPOLI‐1 trial, which investigated treatment with nanoliposomal irinotecan +5‐FU in patients pretreated with gemcitabine and showed a survival advantage over 5‐FU alone (6.2 vs. 4.2 months, HR, 0.75; 95% CI, 0.57–0.99).^8^ Our data are slightly better with a mOS in the second line of around 8 months for patients receiving any combination treatment. However, gemcitabine as standard first‐line treatment as in the NAPOLI trial is not standard for all patients anymore. Same holds true for the 2014 CONKO‐003 trial that also included gemcitabine‐pretreated patients only and established an oxaliplatin‐based second‐line treatment.^13^

In the real‐world Association des gastro‐entérologues oncologues (AGEO) study, a gemcitabine‐based second‐line treatment was analyzed retrospectively after FOLFIRINOX failure, which more realistically reflects the current treatment landscape. Here, a mPFS of 3.5 months and a mOS of 7.1 months were seen for patients treated with gemcitabine in combination with nab‐paclitaxel after first‐line FOLFIRINOX^14^; a result that was confirmed in a recent meta‐analysis, where the combination of gemcitabine and nab‐paclitaxel also outperformed the monotherapy with gemcitabine after first‐line FOLFIRINOX treatment.^15^, ^16^

We specifically analyzed patients who received non‐canonical treatment sequences, specifically gemcitabine/nab‐paclitaxel as first line and NalIri/FOLFIRI as second‐line treatment as well as modified fluorouracil leucovorine, irinotecan, oxaliplatin (mFOLFIRINOX) in the first line and gemcitabine/nab‐paclitaxel in the second line. Interestingly, while no formal statistical comparison was carried out, there was a small numerical benefit for patients receiving the second sequence. However, this can only be hypothesis‐generating and should be formally tested in a prospective trial. We consider a selection bias toward fitter patients in the second sequence as the most likely reason for the difference observed here.

QoL was prospectively measured in our registry via EORTC QLQ‐C30 questionnaires. For patients with palliative chemotherapy, the mean GHS/QoL score at baseline was 46.8 ± 22.53 in the first line situation and 48.6 ± 20.67 in the second‐line situation. These mean baseline GHS/QoL scores indicate that the QoL of patients with newly diagnosed PCA scheduled to receive first‐line therapy in the real‐life setting is severely impaired, compared to a mean GHS/QoL score of 71.5 in the general German adult population.^17^ However, the baseline GHS/QoL scores in patients before first and before second‐line treatments are comparable, indicating that no major decline in QoL is noted in patients that are fit enough to receive second‐line treatment—which is a minority with 45% of all first‐line patients. Predictive variables for proceeding from first to second‐line therapy were mainly reasons for ending first‐line treatment with the patient's wish being the most prominent one, followed by toxicity and progressive disease. This underscores the importance of shared decision‐making with patients and caregivers at all major checkpoints of the disease, which needs to take all aspects of the disease into account.^18^

TTD of QoL in first line patients was concordant with the median PFS. In second line, surprisingly, TTD of QoL appeared to be about double as long as the mPFS. Our most likely explanation, considering the number of completed questionnaires, is that supposedly only patients that did not quit treatment early (e.g., due to progression or toxicity) completed a subsequent questionnaire in second line and were included in this analysis. Therefore, a certain bias with overrepresentation of patients with a more favorable course of disease cannot be excluded.

Comparison of the Global Health Status between the patients receiving (m)FOLFIRINOX and gemcitabine/nab‐paclitaxel in first line showed no substantial difference, neither at the beginning of treatment nor over time. There is no prospective data including western patients comparing both first‐line regimens over time in terms of QoL. The JCOG1407 trial directly compared both regimens, but solely in patients with locally advanced PCA and hence a different focus; also, QoL data were not reported.^19^

When analyzing single items from patients in our registry, those treated with (m)FOLFIRINOX showed better scores over time for physical functioning, appetite loss, and fatigue when compared to patients treated with the doublet. Low patient numbers and missing data hamper interpretation here. Patients treated with the triplet are generally younger and fitter; however, since the baseline score for Global Health Status does not substantially differ between the groups, one might argue that there is a certain influence of the treatment itself.

In the registrational MPACT trial, QoL was not measured. The registrational trial for (m)FOLFIRINOX by the PRODIGE study group did not report absolute scores for QoL; however, TTD of QoL was significantly longer in the triplet group as compared to gemcitabine mono.

Our study's major limitation is the short follow‐up, which clearly limits the robustness of the results. Further projects should be designed to allow for longer follow‐up for all patients to increase the data quality.

In summary, we were able to collect and analyze real‐world data on patients with PCA in Germany treated in a wide range of institutions and show in detail patient flows throughout all further therapy lines. This data will help to further understand the major clinical checkpoints of the disease by providing insights on the areas of greatest need for improvement, such as effective neoadjuvant treatments.

## AUTHOR CONTRIBUTIONS

**Thorsten O. Goetze:** Conceptualization; investigation; funding acquisition; writing – original draft; writing – review and editing; data curation; supervision; methodology. **Salah‐Eddin Al‐Batran:** Conceptualization; investigation; funding acquisition; writing – original draft; writing – review and editing; resources; supervision; project administration; methodology. **Ruediger Liersch:** Writing – review and editing; investigation. **Lars Scheuer:** Investigation; writing – review and editing. **Thomas Goehler:** Writing – review and editing. **Ulrich Kaiser:** Investigation; writing – review and editing. **Claudio Denzlinger:** Writing – review and editing; investigation. **Stephan Brandl:** Investigation; writing – review and editing. **Daniel Pink:** Investigation; writing – review and editing. **Jens Uhlig:** Investigation; writing – review and editing. **Michael Maasberg:** Investigation; writing – review and editing. **Maria Loose:** Investigation; writing – review and editing. **Marina Schaaf:** Writing – review and editing; methodology; validation; visualization; software; formal analysis; data curation; supervision. **Disorn Sookthai:** Software; formal analysis; data curation; visualization; methodology; validation; writing – review and editing. **Bianca Zäpf:** Writing – original draft; writing – review and editing; visualization; methodology; validation; project administration; data curation; supervision. **Claudia Pauligk:** Writing – original draft; writing – review and editing; methodology; validation; visualization; project administration; supervision; data curation. **Timursah Habibzade:** Investigation; writing – review and editing. **Ralf‐Dieter Hofheinz:** Conceptualization; investigation; funding acquisition; writing – review and editing; supervision; methodology. **Christine Koch:** Writing – original draft; writing – review and editing; data curation; methodology; conceptualization.

## CONFLICT OF INTEREST STATEMENT

Salah‐Eddin Al‐Batran reports research funding from Celgene, Lilly, Sanofi, German Cancer Aid (Krebshilfe), the German Research Foundation, the Federal Ministry of Education and Research of Germany, Roche, Eurozyto, Immutep, Ipsen, Bristol‐Myers Squibb, Merck Sharp & Dohme, and AstraZeneca; advisory board membership for Bristol‐Myers Squibb, Merck Sharp & Dohme, and Ely Lilly Germany; and speaker's fees from MCI Deutschland GmbH; ownership interest and CEO of Frankfurt Institute of Clinical Cancer Research (IKF). Christine Koch reports speaker's fees from MSD, DGVS, Bristol‐Myers Squibb, MCI Deutschland GmbH, AstraZeneca, Servier, and Incyte; and meeting support from Ipsen, Merck, Roche, and AstraZeneca. Thorsten O. Goetze reports consulting fees from Amgen, AstraZeneca, Bayer, BMS, Daiichi Sankyo, Foundation Medicine, Lilly, MCI, MSD Sharp & Dohme, Novartis, Roche, Sanofi Aventis, Servier, Deciphera, Boehringer Ingelheim, GSK, research funding from Lilly, AstraZeneca, Incyte, German Research Foundation (DFG), Gemeinsamer Bundesausschuss, German Cancer Aid (Deutsche Krebshilfe), Hector‐Stiftung; Servier and other financial relationships with Amgen, AstraZeneca, BMS, Lilly, Merck Serono, Roche, Sanofi Aventis, Servier, Pierre‐Fabre. Daniel Pink declares institutional fees (outside the submitted work; receipt of grants, research support, honoraria or consultation fees, participation in a company‐sponsored speaker's bureau) from Boehringer Ingelheim, Bristol‐Myers Squibb, PharmaMar, Recordati, Deciphera, Lilly, Roche, Blueprint Medicines. The other authors have no conflicts of interest to declare.

## ETHICS STATEMENT

The trial was conducted according to the Declaration of Helsinki, the Good Clinical Practice guidelines of the International Conference on Harmonization, and relevant German and European laws and directives. Written informed consent was provided by all participants before enrollment. In accordance with German regulations concerning observational studies, patients provided informed consent that was approved by the responsible ethics committee (Landesaerztekammer Hessen, file number FF18/2019, September 23rd, 2019). The trial was published under http://www.clinicaltrials.gov (NCT 04119362).

## Data Availability

Data can be made available and further information is available from the corresponding author upon reasonable request.
